# Ecosystems and ordering: A dataset

**DOI:** 10.1016/j.dib.2024.111085

**Published:** 2024-10-30

**Authors:** Cristiana Maglia, Elana Wilson Rowe

**Affiliations:** Norwegian Institute of International Affairs (NUPI), Oslo, Norway

**Keywords:** Ecosystemic politics, International cooperation, Regional governance, Environment

## Abstract

The dataset presented here is connected to the article “Ecosystems and Ordering: Exploring the Extent and Diversity of Ecosystem Governance,” offering data about cooperation initiatives around 221 cross-bordered ecosystems. This sample of cases was selected from a list of 1525 “meta-ecosystems” catalogued by the World Wide Fund for Nature (WWF) and a team of scientists (terrestrial ecosystems, [6]; freshwater ecosystems, [2]; and marine ecosystems, [9]). The 221 ecosystems were selected because they are shared by four or more bordering countries. Departing from this unit of analysis, we researched the cooperative cross-border governance anchored in each ecosystem and categorized each of these based on the level and type of cooperation. In generating this dataset, our coding scheme was designed to also capture cases of non-cooperation: when our search protocol did not result in the identification of any initiative for an ecosystem, the ecosystem was coded as a “zero case.” When we found initiatives connected to the ecosystems, our coding typology specifically classified cooperation initiatives along two dimensions: cooperation geographical scope and cooperation scope (single or multi-issue). The dataset presents ecosystem-anchored cooperation initiatives, as well as wider initiatives that may address ecosystem issues, to systematically attend to the question of the extent to which and in which form ecosystems are addressed in transboundary governance efforts. The dataset allows for further study of ecosystemic governance patterns, enabling analysis of the causes and consequences of cooperation, since it can be easily integrated with both the ecosystem and state-level data. The dataset is presented in two .csv files and has been handled with R software in order to present the visualization.

Specifications Table

**Table:** 

Subject	Social Sciences – Geography.
Specific subject area	The dataset compiles transnational political efforts around ecosystems, bringing together areas such as Social Sciences, International Relations, and Social Geography.
Data format	.csv file (Raw, analysed, and filtered)
Type of data	Tables, charts, graphs, figures, maps
Data collection	The data was collected through desk research, gathered from various sources, including publicly accessible international cooperation initiatives websites. The raw data is securely stored in the databases of the Norwegian Institute of International Affairs and at Harvard Dataverse.
Data source location	Norwegian Institute of International Affairs (NUPI), Oslo, Norway
Data accessibility	Repository name: Harvard Dataverse Data identification number: 10.7910/DVN/DY4RFS Direct URL to data: https://dataverse.harvard.edu/dataset.xhtml?persistentId=doi:10.7910/DVN/DY4RFS
Related research article	Cristiana Maglia, Elana Wilson Rowe, Ecosystems and Ordering: Exploring the Extent and Diversity of Ecosystem Governance, Global Studies Quarterly, Volume 3, Issue 2, April 2023, ksad028, 10.1093/isagsq/ksad028.

## 1 Value of the Data

• This dataset is built upon a list of ecosystems identified in another study on the basis of natural science criteria and adds information about the different ways these ecosystems are addressed in transnational governance efforts, including the identification of ecosystems that are not formally included in any transnational cooperative effort. This prompts a discussion on potential cooperative strategies among countries regarding their shared ecosystems.
• The dataset goes beyond the scholarly work on regional environmental institutional governance, by having ecosystems themselves as its starting point and unit of analysis, rather than starting from a given pool of environmental treaties, organizations or institutions. This allows for a more systematic assessment of variation in levels and types of ecosystemic governance, including attention to cases in which there is no cooperation.
• Other researchers would be able to reuse the data to further study the patterns of governance across ecosystems here systematized. Having this spectrum of variation in the extent of cooperation to non-cooperation opens up further possibilities in examining causes and consequences of cooperation. The comprehensiveness of our universe of cases gives a baseline for multivariate analysis at the ecosystem and state levels to examine the reasons behind the variations in forms of cooperation and the absence of them.
• The dataset is coded so that it can easily be integrated with additional information on ecosystems (such as the economic services potential of its natural resources or its levels of preservation) as well as state-level data on bordering and cooperation initiatives member states (e.g., income, regime type, and capability, as well as from the relations among neighbors, e.g., alliances, conflicts, and comembership in organizations).

## 2 Background

Our research expands upon the scholarship of environmental governance cooperation analysis, moving beyond the conventional analysis in the discussion of ecosystems. The dataset aimed to depart from the typical focus on environmental institutions literature by having ecosystems as the unit of analysis. This rationale allowed us to explore whether and how ecosystems are governed, rather than taking already successful and operating environmental institutions, organizations and treaties as a baseline. Having ecosystems as a starting point and unit of analysis allows for a new window into varying forms and levels of governance across these pieces of nature. Our dataset analyzes the range of transnational governance anchored in ecosystems, differentiating cooperation initiatives as geographically anchored or in broader multilateralism; and as to whether they were focused on specific functional policy fields or on a wider suite of governance challenges. We also identify cases of noncooperation, where states do not construct any international arrangement to govern their shared ecosystems. We argue that this broader set of cases deserves greater attention in international relations to better account for the diversity in global ordering practices. By focusing on an often overlooked object of governance [3,5], we aim to explore the potential universe of cases for ‘ecosystemic politics’ and the broader ordering implications and possibilities that stem from anchoring governance in transboundary ecosystems [10].

## 3 Data Description

The dataset systematizes information about political international cooperation efforts anchored in ecosystems. It is thus built upon the investigation of a wide range of cross-border ecosystems to classify the level and type of cooperation among states in those areas. No doubt, ecosystems and their contours are of enduring interest. The multiple methods of ecosystem classification are reflected in both research and public policies. In this article, the selection of ecosystems was based on the World Wildlife Fund (WWF) mapping of terrestrial (terrestrial ecoregions of the world [TEOWs] [6]), marine (marine ecoregions of the world [MEOWs] and marine provinces [9]), and freshwater (freshwater ecoregions of the world [FEOWs] [2]) areas. Their definition proposes that these delimited ecosystems (or ecoregions) are “relatively large units of land or water containing a distinct assemblage of natural communities sharing a large majority of species, dynamics, and environmental conditions.” This natural science criterion and particular catalog of ecosystems is also used by other scholars. A recent example is the work by Shawky et al. [8] which uses the classification of the same group of scientists [4], to research the relationship between land coverage and temperature in the South Asia ecoregions.

We have used the entries identified by this team of scientists (867 terrestrial ecosystems, 232 marine ecosystems, 426 freshwater ecosystems, and 62 larger marine provinces) to select the sample of cases that we included in the dataset. Because we were particularly interested in the complex cooperation around these ecosystems and to make the hand coding protocol more feasible, we limited the population of ecosystems to researching only those that had four or more bordering countries: 221 ecosystems.^1^

It is worth noting that our case selection criterion allows us to capture complex scenarios of ecosystems adjacent to multiple countries, but also limits our geographical range since the global distribution of ecosystems is uneven. Europe and Africa exhibit particularly high levels of potential terrestrial governance complexity because more ecosystems are shared by four or more states in those regions. This is also the case for freshwater ecosystems, where Europe and Africa, followed by Central Asia and South America, have more ecosystems cutting across for or more states. For marine ecosystems, the Caribbean, Europe, and Middle East/North Africa have most ecosystems with multiple adjacent states. In contrast, important geographical areas such as the South Asia region, with high population density and significant ecological challenges [8], are not included in our analysis due to this selection criterion. Expanding the analysis to ecosystems shared by smaller numbers of countries (two or three) will be an important avenue for future scholarship to extend this data set.

Following our protocol, we further researched and categorized the cooperative cross-border governance initiatives anchored in each ecosystem. To ensure that we captured a comprehensive range of them, we searched for various kinds of cooperation efforts, such as intergovernmental organizations, integration processes, cooperation treaties (even those that did not get formally institutionalized), formal environmental institutions, strategies, and projects within international organizations, and informal international organizations.

We classified and differentiated initiatives along two dimensions: *cooperation geographical scope* and *cooperation focus*.

- The first dimension distinguishes cooperation initiatives that were specifically tied to an ecosystem from those broader multilateral ones whose mandate includes the ecosystem.
- The second dimension differentiates initiatives focusing on a broader array of issues and themes from those that only include specific topics (i.e., environment).

**Fig. 1:**
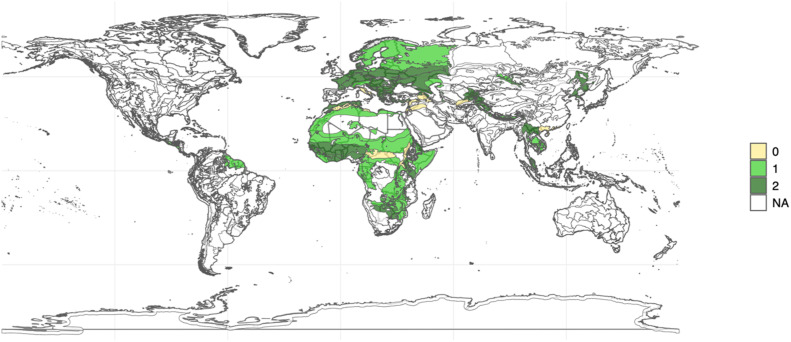
Map of Terrestrial Ecosystems coded considering cooperation initiatives. Source: The map in Fig. 1 was produced using the R software, with shapefiles collected from the WWF website (available at: https://www.worldwildlife.org/publications/terrestrial-ecoregions-of-the-world). Caption: map of terrestrial ecosystems with four or more adjacent countries with geographically specific organizations (1, light green), with only broader initiatives (2, dark green) and without cooperation initiatives (0, yellow). (For interpretation of the references to color in this figure, the reader is referred to the web version of this article.)

**Fig. 2:**
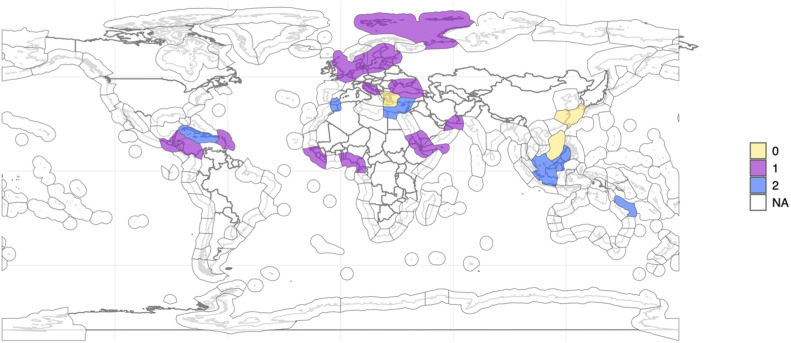
Map of Marine Ecosystems coded considering cooperation initiatives. Source: The map in Fig. 2 was produced using the R software, with shapefiles collected from the WWF website (available at: https://www.worldwildlife.org/publications/marine-ecoregions-of-the-world-a-bioregionalization-of-coastal-and-shelf-areas). Caption: map of marine ecosystems with four or more adjacent countries with geographically specific organizations (1, purple), with only broader initiatives (2, blue) and without cooperation initiatives (0, yellow). (For interpretation of the references to color in this figure, the reader is referred to the web version of this article.)

**Fig. 3:**
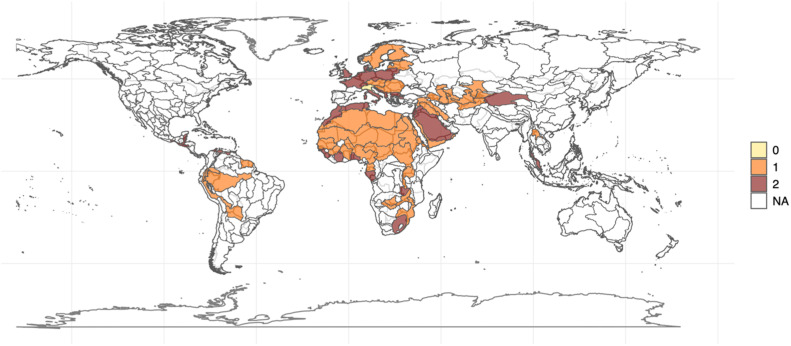
Map of Freshwater Ecosystems coded considering cooperation initiatives. Source: The map in Fig. 3 was produced using the R software, with shapefiles collected from the WWF website (available at: https://www.feow.org/download). Caption: map of freshwater ecosystems with four or more adjacent countries with geographically specific organizations (1, orange), with only broader initiatives (2, brown) and without cooperation initiatives (0, yellow). (For interpretation of the references to color in this figure, the reader is referred to the web version of this article.)

When we could not find any cooperation initiatives around the ecosystem, we coded the ecosystem as a case of non-cooperation. The coding for the types of ecosystem cooperation was a product of the cooperation initiatives relevant to the ecosystems. If the ecosystem hosts at least one specific cooperation initiative, we assigned it a code of 1; for ecosystems associated only with broader cooperation initiatives, we coded it 2. In the map in Fig. 1, we can see terrestrial ecosystems with four or more adjacent countries that have geographically specific organizations (assigned as 1, in light green), those in which their adjacent countries participate only in broader initiatives (assigned as 2, in dark green) and cases of non-cooperation (assigned as 0, in yellow). In the map in Fig. 2, the same rationale is applied to marine ecosystems, with ecosystems with geographically specific organizations as 1 (purple), with only broader initiatives as 2 (blue), and non-cooperation coded as 0 (yellow). Fig. 3 applies the same approach for freshwater ecosystems: those with specific cooperation initiatives as 1 (orange), with broader organizations as 2 (brown), and with non-cooperation as 0 (yellow).

Because of this coding strategy, our dataset allows ecosystems to have multiple initiatives anchored on each of them and for the same initiatives to be anchored in multiple ecosystems. Yet, our unit of analysis is the unique relationship between an ecosystem and a cooperation initiative. In order to best present the information in this dataset, we assembled two different CSV files. The first one (“wide” format: dataset_ecosystem_wide.csv) has each one of the ecosystems as an observation and gathers information on them and the cooperation initiatives connected to them. The first ten columns present general information on the ecosystem level (such as id, biome, realm, and adjacent countries), whereas the remaining columns contain information about the initiatives anchored in that ecosystem. We identified up to five different initiatives by ecosystem. On each of them, we gathered the following information: title, year of establishment, list of adjacent countries, percentage of the countries in the ecosystem that are part of the cooperation, percentage of the countries that are members in the cooperation that are part of the ecosystem (to cover the overlap between adjacent countries and members on initiatives), the address of the secretariat, description of the cooperation, and four columns regarding the code that result in the type of the cooperation initiative, considering the two dimensions (geographical scope and cooperation focus). The same structure follows for each cooperation initiative: Cooperation 1 to Cooperation 5, resulting in 65 columns.

**Fig. 4:**
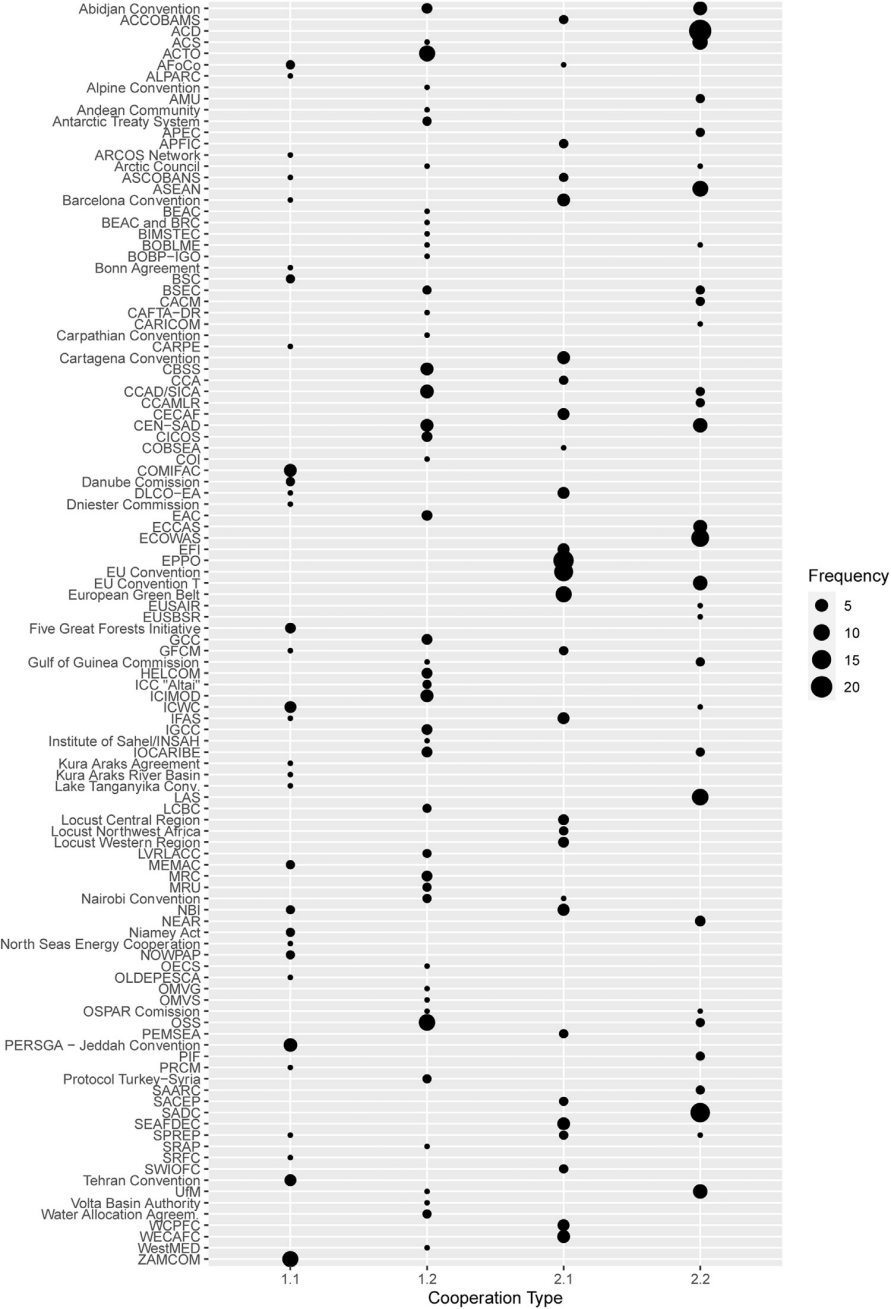
Cooperation Initiatives by frequency.

The second file is the database in a “long” format (dataset_ecosystem_initiatives_long.csv), with cleaned and rearranged data in order to focus on the cooperation initiatives as the units of analysis, even though keeping data in combination with the ecosystem in which they are anchored (such as the percentage of overlap between the states in the ecosystem and in the cooperation initiative). Fig. 4 presents the list of initiatives coded by frequency according to our classification rationale, presented in Maglia & Rowe [1]. Classification 1.1 refers to specific cooperation initiatives, in both geographical scope and cooperation focus. Classification 1.2 applies to those that are geographically specific and broader in cooperation issue focus. Classification 2.1 includes initiatives that are broader geographically and issue-specific. Classification 2.2 pertains to those that are broad in both geographical and cooperation scopes.

As the data collection required information on cooperation initiatives anchored in those ecosystems, the database has the potential for further research regarding ecosystem institutions.

## 4 Experimental Design, Materials and Methods

The identification of the level of international ecosystemic cooperation in each ecosystem followed four-step protocol. The first step was the identification of keywords from the WWF and the Wikipedia pages about the ecosystem – considering the name of the ecosystem and its characteristic geography features, such as a river, mountain, forest, sea, gulf that was central to the ecosystem. The second step was to search for these ecosystems and keywords in relevant databases: Correlates of War codebook of International Organizations [7],^2^ the International Environmental Agreements Database (IEADB),^3^ the Yearbook of International Organizations of the Union of International Associations,^4^ and the UN Treaty Collection.^5^ For the MEOWs and larger marine provinces, the search of the keywords also comprised the Large Marine Ecosystems (LME) databases^6^ and the Regional Fishery Bodies (RFB) of the FAO Fisheries Division database.^7^ The third step was to search more broadly on Google for initiatives regarding *cooperation, sustainable development, conservation, protection* in each of the ecosystems, also according to the keywords gathered in the first step. The fourth step was to search for regional integration processes of which some of the countries in the ecosystem were part and which could harbour cooperation among the states in each ecosystem. For each cooperation initiative, we collected information on member countries, the year of establishment, where it is based, and a general description collected on their websites. We collected data for up to five initiatives per ecosystem.

## Limitations

Our dataset is constrained by the limited sample of 221 ecosystems. Our decision to restrict the scope stemmed from our particular interest in the complex cooperation around cross-bordered ecosystems. Although there was a potential to research ecosystems shared by three countries or more, we opted for a narrowed focus to make the hand-coding protocol feasible. This resulted in a geographical unevenness, with few cases in some continents, such as Asia and North America. Future research aims to expand the dataset to include these additional cases of ecosystems shared by two or three countries. Another possible extension could be to look at additional cooperation efforts for ecosystems that have more than five initiatives anchored on them.

While collecting the data, we encountered the anticipated challenge of mismatches between the political and natural science classifications of ecosystems. To give some examples, the Mediterranean is divided into 8 entries in the WWF classification, and the Sahel and the Sahara into 7 entries. This resulted in cooperation initiatives such as the Sahara and Sahel Observatory (OSS) and the Amazon Cooperation Treaty Organization (ACTO) being coded as specific for geographic scope in more than one ecosystem /entry.

## Ethics Statement

Not applicable. These data do not include data from experiments with any human subjects, animal experiments, or social media platforms.

## Credit Author Statement

**Cristiana Maglia:** Methodology, Data curation, Visualization, Writing – original draft, Writing – review & editing; **Elana Wilson Rowe:** Conceptualization, Methodology, Validation, Writing – original draft, Writing – review & editing, Project administration, Funding acquisition.

## Data Availability

DataverseEcosystems and Ordering: Exploring the Extent and Diversity of Ecosystem Governance (Original data).

## Footnotes

1 For clarity, ecosystems contained within a single country comprise 496 TEOWs, 149 MEOWs, 20 larger marine provinces, and 201 FEOWs. Those shared by two countries include 197 TEOWs, 37 MEOWs, 6 marine provinces, and 103 FEOWs. Ecosystems with three adjacent countries encompass 79 TEOWs, 18 MEOWs, 9 marine provinces, and 60 FEOWs.

2 Pevehouse, Jon C.W., Timothy Nordstron, Roseanne W McManus, Anne Spencer Jamison, “Tracking Organizations in the World: The Correlates of War IGO Version 3.0 datasets”, Journal of Peace Research. Available at: <https://correlatesofwar.org/data-sets/IGOs>.

3 Available at: <https://iea.uoregon.edu>.

4 Available at: <https://uia.org/ybio/>.

5 Available at: <https://treaties.un.org>.

6 Large Marine Ecosystems Hub website, available at: <https://www.lmehub.net>. GEF International Waters project portfolio website, available at: <https://iwlearn.net/iw-projects/>. Transboundary Waters Assessment Programme (TWAP) website, available at: <http://onesharedocean.org/lmes>. Marine Regions website, available at: <https://www.marineregions.org/>.

7 Available at: <http://www.fao.org/fishery/rfb/en>
